# Influence of *IL‐1R2* polymorphisms on endometrial cancer susceptibility in the Chinese Han population

**DOI:** 10.1002/mgg3.650

**Published:** 2019-03-20

**Authors:** Jiamin Wu, Wenjie Zhang, Junhong Cai, Sizhe Huang, Fanglin Niu, Ying Zhang, Shan Bao, Tianbo Jin

**Affiliations:** ^1^ Key Laboratory of Resource Biology and Biotechnology in Western China, Ministry of Education Northwest University Xi’an Shaanxi China; ^2^ Department of Women’s Health Care Northwest Women and Children Hospital Xi'an Shaanxi China; ^3^ Key Laboratory of Cell and Molecular Genetic Translational Medicine in Hainan Province Hainan General Hospital Haikou Hainan China; ^4^ Department of Gynaecology and Obstetrics Hainan General Hospital Haikou Hainan China; ^5^ Key Laboratory for Basic Life Science Research of Tibet Autonomous Region, School of Medicine Xizang Minzu University Xianyang Shaanxi China

**Keywords:** endometrial cancer, *IL‐1R2*, polymorphisms, susceptibility

## Abstract

**Background:**

Recently, many studies have identified that genetic factor plays a crucial role in endometrial cancer development. The purpose of this study is to investigate the influence of single nucleotide polymorphisms (SNPs) of *IL‐1R2 *on endometrial cancer susceptibility.

**Methods:**

We performed a case‐control study that included 293 patients with endometrial cancer and 579 healthy controls. Six SNPs in the *IL‐1R2* gene were genotyped using the Agena MassARRAY platform. Genetic models and haplotype analyses were used to assess the association between SNPs and endometrial cancer risk by computing odds ratios (ORs) and 95% confidence intervals (CIs).

**Results:**

Overall analysis results found that two SNPs (rs4851527 and rs3218896) and haplotypes TGTC and TACT were significantly associated with endometrial cancer risk. Stratified analysis by age showed that rs2072472 was associated with endometrial cancer risk in age >54 subgroup.

**Conclusions:**

These findings suggested that *IL‐1R2 *polymorphisms may contribute to the development of endometrial cancer. Further studies are required to confirm the results.

## 1 INTRODUCTION

Endometrial cancer is the most common type of gynecological malignancy worldwide and has a large geographic variation in incidence and mortality rates (Bray et al., 2018). In China, there has been a significant increase in the number of women diagnosed with endometrial cancer (Chen et al., 2016). Although early menarche, unopposed estrogen, endometriosis, late menopause, obesity, diabetes, hypertension, and nulliparity are well‐known risk factors for the development of endometrial cancer (Amant et al., 2005), only a part of individuals exposed to these risk factors develop endometrial cancer during their lifetime, suggesting that genetic factor plays a crucial role in endometrial cancer development. The single nucleotide polymorphism (SNP) is the most common form of human genetic variations. It has been reported that polymorphisms of interleukin genes are significantly associated with gynecologic cancers risk (Koensgen et al., 2015; Yu et al., 2015; Zhou et al., 2018). Recently, genome‐wide association analysis studies have identified many risk loci in the candidate endometrial cancer susceptibility genes, such as *HNF1B* (Painter et al., 2015), *LOC643623*, *AKT1*, and *KLF5* (Burki, 2016; Cheng et al., 2016). However, the mechanism of endometrial cancer is still unclear.

The association between inflammation and cancer have been recognized for some years and recently become the focus of tumor studies (Grivennikov, Greten, & Karin, 2010). It has also indicated the proinflammatory milieu can directly increase estrogen production, which may facilitate carcinogenesis by disrupting the estrogen‐progesterone balance (Modugno, Ness, Chen, & Weiss, 2005). A growing number of studies reported that SNPs locus in interleukin (IL) genes, such as *IL‐32* (Yu et al., 2015), *IL‐6* (Wang, Zhang, Zheng, Liu, & Li, 2016), and *IL1A* (Yu et al., 2016), are associated with the risk endometrial cancer. Interleukin 1 receptor type 2 (IL‐1R2) is located on the long arm of human chromosome 2 at band 2q12, belongs to the interleukin 1 receptor family (Boraschi & Tagliabue, 2013). *IL‐1R2 *serves as a negative regulator of *IL‐1* signaling by competing with *IL‐1R1 *for *IL‐1* and by complexing with *IL‐1* receptor accessory protein (IL‐1RAP) once it binds *IL‐1*, thereby sequestering both the ligand and the accessory protein required for signal transduction (Lang et al., 1998). *IL‐1R2* is an important mediator involved in many cytokine induced immune and inflammatory responses (Peters, Joesting, & Freund, 2013).

Association studies between *IL‐1R2 *gene polymorphisms and diseases have been carried out recently (Ren, Dong, Huyan, Jin, & Chen, 2018; Xia et al., 2015; Xie et al., 2017). However, the influence of *IL‐1R2 *polymorphisms on endometrial cancer susceptibility in the Chinese Han population has not been reported yet. Given the role of *IL‐1R2* in immune regulation and inflammatory response, we hypothesized that common genetic polymorphisms in the *IL‐1R2* gene may also influence the risk of endometrial cancer. To investigate this hypothesis, we recruited 293 patients with endometrial cancer and 579 healthy controls to investigate the association between polymorphisms in the *IL‐1R2* gene and endometrial cancer risk in the Chinese Han women population.

## 2 MATERIALS AND METHODS

### 2.1 Study participants

In this case‐control study, a total of 293 female patients with new diagnosis of endometrial cancer were recruited from the Hainan General Hospital and the Northwest Women and Children Hospital. All cases were confirmed histologically to have endometrial cancer. The patients were recruited without restrictions of age, sex, or disease stage. The controls were 579 females randomly selected from a pool of healthy volunteers who visited the general health check‐up center at the same hospitals during the same period. The mean age of the participants was 48.06 years in the control group and 59.31 years in the case group, respectively. Women who have a history of any cancer or hysterectomy were excluded in the study. The case and control subjects were Chine Han population.

This study was performed in accordance with the ethical principles of the Declaration of Helsinki and was approved by the Ethics Committee of the Hainan General Hospital and the Northwest Women and Children Hospital. All of the participants voluntarily agreed to participate in this study and all provided written informed consent.

### 2.2 Genotyping

We collected 5ml peripheral blood samples from each subject using venipuncture into ethylene diamine tetraacetic acid (EDTA)‐coated blood vacutainer collection tubes and then stored at −80°C for further use. We used the GoldMag‐Mini Whole Blood Genomic DNA Purification Kit (GoldMag. Co. Ltd., Xi'an, China) to extract genomic DNA from blood samples following the manufacturer's instructions. We assessed the purity and concentration of the extracted DNA using a spectrophotometer (NanoDrop 2000; Thermo Fisher Scientific, Waltham, MA) by absorbance measurements at 260 and 280 nm.

Six SNPs (rs11674595, rs4851527, rs719250, rs3218896, rs3218977, and rs2072472) in *IL‐1R2 *with minor allele frequency (MAF) greater than 0.05 in the global population from the HapMap database and previously reported were adopted for analysis. We used the Agena Bioscience Assay Design Suite V2.0 software (https://agenacx.com/online-tools/) to design the primers of PCR amplification and extension of the six selected SNPs. These SNPs in *IL‐1R2 *were genotyped in the case and control groups using the Agena MassARRAY platform with iPLEX gold chemistry (Agena Bioscience, San Diego, CA) according to the manufacture's instructions. We used the Agena Bioscience TYPER software (version 4.0) to manage and analyze data.

### 2.3 Statistical analysis

The Hardy–Weinberg equilibrium (HWE) was performed for each polymorphism among controls using the PLINK software (version 1.07) (Purcell et al., 2007). We compared the distributions of SNPs allele and genotype frequencies between cases and controls using *χ* ^2^ test. The association analyses were conducted using logistic regression analysis under codominant, dominant, recessive, and additive genetic models with adjustment for age. Pair‐wise linkage disequilibrium (LD) between the five SNPs was assessed using the Haploview software (version 4.2) (Barrett, Fry, Maller, & Daly, 2005). Odds ratios (ORs) and 95% confidence intervals (CIs) were calculated to measure the potential association between SNPs and endometrial cancer risk (Lin et al., 2017; Tian et al., 2018). *p* value less than 0.05 was considered statistically significant. All statistical tests were two‐sided. The statistical analyses were performed using the Statistical Package of the Social Sciences (SPSS) software version 20.0 (SPSS Inc., Chicago, IL).

## 3 RESULTS

The distributions of the genotype frequency of the six SNPs among the healthy controls were found to be in accordance with the HWE (*p* > 0.05). We used Pearson *χ* ^2^ test to compare the distributions of the allele frequency of the SNPs in *IL‐1R2* between the case group and the control group (Table 1). However, the allele frequency of all the six SNPs in case group did not differ significantly compared to that in the control group (*p* > 0.05). There was no statistically significant association between the *IL‐1R2 *polymorphisms and endometrial cancer risk in the Chinese Han population.

**Table 1:** Alleles distribution of *IL‐1R2 *polymorphisms and association with endometrial cancer risk

SNP‐ID	Chr	Position	Alleles A/B	MAF		HWE‐*p*	OR (95%CI)	*p*
				Case	Control			
rs11674595	2	101994530	C/T	0.222	0.222	0.718	1.00 (0.78–1.27)	0.978
rs4851527	2	102005914	A/G	0.265	0.307	0.284	0.81 (0.65–1.01)	0.063
rs719250	2	102007256	A/G	0.319	0.301	0.201	1.09 (0.88–1.35)	0.448
rs3218896	2	102015190	C/T	0.166	0.136	0.596	1.27 (0.96–1.67)	0.091
rs3218977	2	102024739	A/G	0.249	0.225	0.095	1.15 (0.91–1.45)	0.252
rs2072472	2	102026557	C/T	0.240	0.216	0.326	1.15 (0.90–1.45)	0.260

Note

95% CI: 95% Confidence interval; A: Minor allele; B: Major allele; Chr: chromosome; HWE: Hardy Weinberg equilibrium; MAF: Minor allele frequency; OR: Odds ratio; SNP: Single nucleotide polymorphism.

*p* values were calculated from *χ* ^2^ test ( two sided).

*p < *0.05 was considered statistically significant.

Next, we further evaluated the association between the *IL‐1R2 *polymorphisms and endometrial cancer risk under the genetic models (codominant, dominant, recessive, and additive) by logistic regression analysis adjusting for age (Table 2). Compared with the GG wild‐type homozygous genotype, the AG genotype of rs4851527 was associated with a decreased risk of endometrial cancer (OR = 0.71, 95% CI: 0.52–0.96, *p* = 0.028). When the wild‐type homozygous genotype GG was used as a reference, the variant homozygote AA and heterozygote AG genotypes were also found to be associated with a reduced risk of endometrial cancer in the dominant model (OR = 0.71, 95% CI: 0.53–0.96, *p* = 0.024). Similar association was found between rs4851527 and the risk of endometrial cancer in the additive model (OR = 0.79, 95% CI: 0.63–1.00, *p* = 0.047).

**Table 2:** Genetic model analyses of the association between *IL‐1R2* polymorphisms and endometrial cancer risk

SNP–ID	Model	Genotype	Case	Control	Without adjust OR (95%CI)	*p*	Adjust OR (95%CI)	*p*
rs11674595	Codominant	TT	182	350	1.00		1.00	
		CT	89	196	0.87 (0.64–1.19)	0.388	0.90 (0.65–1.24)	0.515
		CC	20	30	1.28 (0.71–2.32)	0.412	1.21 (0.65–2.25)	0.548
	Dominant	TT	182	350	1.00		1.00	
		CC + CT	109	226	0.93 (0.69–1.24)	0.612	0.94 (0.70–1.27)	0.699
	Recessive	TT + CT	271	546	1.00		1.00	
		CC	20	30	1.34 (0.75–2.41)	0.322	1.25 (0.68–2.31)	0.468
	Additive	–	–	–	1.00 (0.79–1.26)	0.979	1.00 (0.78–1.27)	0.977
rs4851527	Codominant	GG	163	272	1.00		1.00	
		AG	105	258	0.68 (0.50–0.92)	0.011	0.71 (0.52–0.96)	0.028
		AA	25	49	0.85 (0.51–1.43)	0.544	0.74 (0.43–1.28)	0.280
	Dominant	GG	163	272	1.00		1.00	
		AA + AG	130	307	0.71 (0.53–0.94)	0.016	0.71 (0.53–0.96)	0.024
	Recessive	GG + AG	268	530	1.00		1.00	
		AA	25	49	1.01 (0.61–1.67)	0.972	0.86 (0.51–1.46)	0.580
	Additive	–	–	–	0.81 (0.65–1.01)	0.063	0.79 (0.63–1.00)	0.047
rs719250	Codominant	GG	137	289	1.00		1.00	
		AG	125	231	1.14 (0.85–1.54)	0.384	1.25 (0.92–1.71)	0.152
		AA	31	59	1.11 (0.69–1.79)	0.674	1.20 (0.73–1.97)	0.470
	Dominant	GG	137	289	1.00		1.00	
		AA + AG	156	290	1.14 (0.86–1.50)	0.379	1.24 (0.93–1.67)	0.145
	Recessive	GG + AG	262	520	1.00		1.00	
		AA	31	59	1.04 (0.66–1.65)	0.858	1.08 (0.67–1.74)	0.745
	Additive	–	–	–	1.08 (0.88–1.34)	0.458	1.15 (0.92–1.43)	0.215
rs3218896	Codominant	TT	203	433	1.00		1.00	
		CT	81	133	1.30 (0.94–1.79)	0.112	1.41 (1.00–1.97)	0.048
		CC	8	12	1.42 (0.57–3.53)	0.448	1.50 (0.58–3.85)	0.401
	Dominant	TT	203	433	1.00		1.00	
		CC + CT	89	145	1.31 (0.96–1.79)	0.091	1.41 (1.02–1.96)	0.037
	Recessive	TT + CT	284	566	1.00		1.00	
		CC	8	12	1.33 (0.54–3.29)	0.539	1.37 (0.54–3.51)	0.509
	Additive	–	–	–	1.26 (0.96–1.66)	0.094	1.34 (1.01–1.79)	0.043
rs3218977	Codominant	GG	163	341	1.00		1.00	
		AG	111	216	1.08 (0.80–1.44)	0.631	1.05 (0.77–1.43)	0.747
		AA	17	22	1.62 (0.84–3.13)	0.154	1.28 (0.65–2.54)	0.475
	Dominant	GG	163	341	1.00		1.00	
		AA + AG	128	238	1.13 (0.85–1.50)	0.417	1.08 (0.80–1.44)	0.628
	Recessive	GG + AG	274	557	1.00		1.00	
		AA	17	22	1.57 (0.82–3.01)	0.173	1.26 (0.64–2.46)	0.505
	Additive	–	–	–	1.16 (0.91–1.47)	0.239	1.09 (0.85–1.39)	0.515
rs2072472	Codominant	TT	174	360	1.00		1.00	
		CT	96	188	1.06 (0.78–1.43)	0.724	1.09 (0.80–1.50)	0.586
		CC	22	31	1.47 (0.83–2.61)	0.191	1.44 (0.79–2.64)	0.232
	Dominant	TT	174	360	1.00		1.00	
		CC + CT	118	219	1.12 (0.84–1.49)	0.459	1.14 (0.85–1.54)	0.382
	Recessive	TT + CT	270	548	1.00		1.00	
		CC	22	31	1.44 (0.82–2.54)	0.206	1.40 (0.77–2.53)	0.265
	Additive	–	–	–	1.14 (0.90–1.43)	0.275	1.15 (0.91–1.46)	0.254

Note

95% CI: 95% Confidence interval; OR: Odds ratio; SNP: Single nucleotide polymorphism.

Adjust OR and 95% CI were calculated using a conditional logistic regression adjusted with age. *p* < 0.05 was considered statistically significant.

Additionally, we observed a statistically significant association between the rs3218896 polymorphism and endometrial cancer risk. The relative OR of 1.41 (95% CI: 1.00–1.97) showed that the variant heterozygote CT was correlated with a higher risk of developing endometrial carcinoma comparing with the wild‐type homozygous genotype TT. A statistically significant interaction was observed between the variant homozygote CC and heterozygote CT genotypes of rs3218896 for an increased risk of endometrial cancer in the dominant model compared with the TT wild‐type genotype individuals (OR = 1.41, 95% CI: 1.02–1.96, *p* = 0.037). We also found that the SNP rs3218896 was significantly associated with an increased risk of endometrial cancer in the additive model (OR = 1.34, 95% CI: 1.01–1.79, *p* = 0.043). However, no associations between the four *IL‐1R2* polymorphisms (rs11674595, rs719250, rs3218977, and rs2072472) and endometrial cancer risk were observed in the different genetic models.

**Figure 1:**
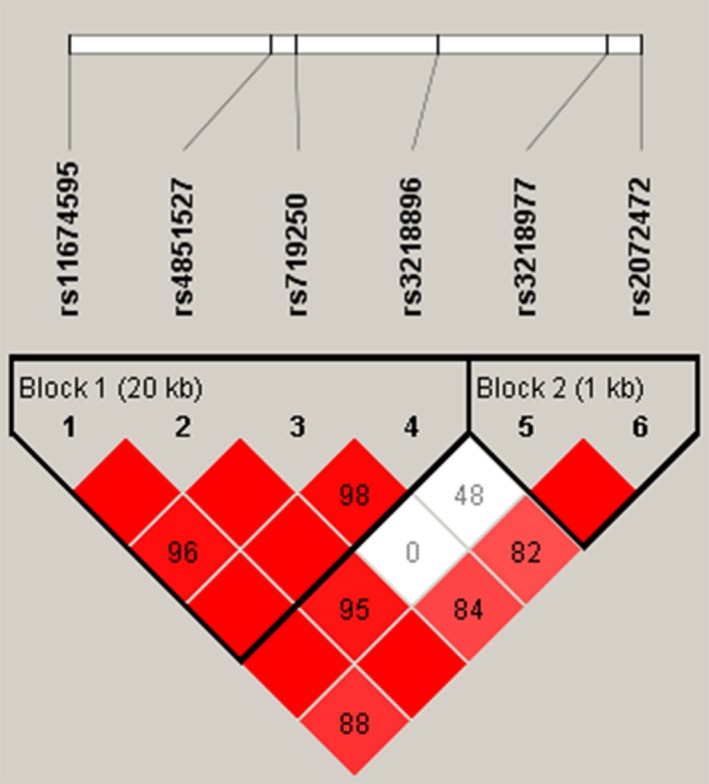
Haplotype block map for the six SNPs in the *IL‐1R2* gene. Pairwise linkage disequilibrium (LD) measures (D and r^2^) were shown by the LD map. Square background color represents the D’/LOD and the values in cells are r^2^ values (multiplied by 100). Bright red represents very strong LD; white represents no LD; pink represents intermediate LD

The results of pair‐wise LD analysis with these six SNPs are shown in Figure 1. We observed two small haplotype blocks; first composed of rs11674595, rs4851527, rs719250, and rs3218896; second of rs3218977 and rs2072472. The distributions of the frequencies of the haplotypes TGTC and TACT were significantly different between endometrial cancer and control groups (*p* = 0.031 and *p* = 0.049, respectively). Logistic regression analysis confirmed that the haplotypes TGTC (OR = 0.73, 95% CI: 0.54–0.97) and TACT (OR = 0.79, 95% CI: 0.63–1.00) were significantly associated with decreased endometrial cancer risk after adjusting for age (Table 3). Moreover, the haplotype AA was found to be significantly associated with a reduced risk of endometrial cancer before adjusting for age (OR = 0.81, 95% CI: 0.67–0.99, *p* = 0.044) (Table 3).

**Table 3:** Association between haplotypes of *IL‐1R2* and endometrial cancer risk

SNP‐ID	Haplotype	FA	FU	OR (95%CI)	*p*	Adjust OR (95%CI)	Adjust *p*
rs11674595|rs4851527|rs719250|rs3218896	TGTC	0.835	0.866	0.78 (0.59–1.03)	0.080	0.73 (0.54–0.97)	0.031
rs11674595|rs4851527|rs719250|rs3218896	TGTT	0.848	0.837	1.09 (0.83–1.44)	0.530	1.04 (0.78–1.38)	0.778
rs11674595|rs4851527|rs719250|rs3218896	TACT	0.266	0.309	0.81 (0.65–1.01)	0.063	0.79 (0.63–1.00)	0.049
rs11674595|rs4851527|rs719250|rs3218896	CGCT	0.778	0.781	0.98 (0.78–1.24)	0.879	0.98 (0.77–1.25)	0.860
rs11674595|rs4851527|rs719250|rs3218896	TGCT	0.807	0.829	0.86 (0.66–1.12)	0.257	0.93 (0.71–1.21)	0.577
rs3218977|rs2072472	AG	0.759	0.784	0.87 (0.69–1.10)	0.244	0.86 (0.68–1.10)	0.227
rs3218977|rs2072472	GA	0.75	0.776	0.86 (0.68–1.10)	0.224	0.92 (0.71–1.17)	0.487
rs3218977|rs2072472	AA	0.509	0.56	0.81 (0.67–0.99)	0.044	0.84 (0.68–1.04)	0.105

Note

95% CI: 95% Confidence interval; FA: Frequency in case; FU: Frequency in control; OR: Odds ratio.

OR and 95% CI were calculated using a conditional logistic regression.

*p* value < 0.05 indicates statistical significance.

Given that age is a major endometrial cancer risk factor, we further evaluated the association between polymorphisms of *IL‐1R2* and endometrial cancer risk by age stratified analysis (Table 4). The CC genotype of rs2072472 was found to be associated with an increased risk of endometrial cancer compared with the TT wild‐type homozygous genotype in age >54 years old subgroup (OR = 2.28, 95% CI: 1.04–5.00, *p* = 0.040). When compared with the variant homozygote TT and heterozygote CT genotypes, the genotype CC was also found to be associated with an increased risk of endometrial cancer in the recessive model in the subgroup of age >54 years (OR = 2.33, 95% CI: 1.08–5.03, *p* = 0.032).

**Table 4:** Age stratification analysis of association between rs2072472 and endometrial cancer risk

Model	Genotype	Age ≤54 years old				Age >54 years old			
		Case	Control	OR (95%CI)	*p*	Case	Control	OR (95%CI)	*p*
Codominant	TT	83	238	1.00		91	122	1.00	
	CT	43	133	0.94 (0.61–1.46)	0.785	53	55	1.30 (0.82–2.08)	0.268
	CC	14	18	2.28 (1.04–5.00)	0.040	8	13	0.77 (0.30–1.96)	0.586
Dominant	TT	83	238	1.00		91	122	1.00	
	CC + CT	57	151	1.10 (0.73–1.65)	0.660	61	68	1.20 (0.77–1.86)	0.422
Recessive	TT + CT	126	371	1.00		144	177	1.00	
	CC	14	18	2.33 (1.08–5.03)	0.032	8	13	0.71 (0.28–1.77)	0.458
Additive	–	–	–	1.22 (0.88–1.69)	0.225	–	–	1.06 (0.75–1.51)	0.729

Note

95% CI: 95% Confidence interval; OR: Odds ratio.

*p* < 0.05 was considered statistically significant.

## 4 DISCUSSION

In this study, we investigate the influence of *IL‐1R2* polymorphisms on endometrial cancer susceptibility in the Chinese Han population. Overall analysis results found that rs4851527 was associated with a decreased risk of endometrial cancer; rs3218896 was significantly associated with an increased risk of endometrial cancer. Haplotype analysis confirmed that the haplotypes TGTC and TACT were significantly associated with decreased endometrial cancer risk. Moreover, stratification analysis showed that rs2072472 was associated with an increased risk of endometrial cancer in age >54 subgroup.

*IL‐1R2* was first characterized by McMahon et al. in 1991 and natively found on neutrophils, B‐cells, monocytes, and macrophages (McMahan et al., 1991). *IL‐1R2* serves as an endogenous inhibitor of *IL‐1* signaling by competing with *IL‐1R1 *for *IL‐1*, and by subsequently forming a complex with IL‐1RAcP, thereby sequestering both the ligand and the accessory protein required for signal transduction (Schluter, Schelmbauer, Karram, & Mufazalov, 2018). Additionally, *IL‐1R2 *exists in both a membrane bound and soluble form (sIL‐1R2) that has biological properties similar to both a decoy receptor and a binding protein (Peters et al., 2013). Study found that *IL‐1R2* regulates the cell metabolism and the response of immune inflammation induced by many cytokines (Dinarello, 1994). These suggest that *IL‐1R2* is an important mediator involved in many cytokine induced immune and inflammatory responses*. *Inflammation aids in the proliferation and survival of malignant cells, promotes angiogenesis and metastasis, subverts adaptive immune responses, and thus initiate and promote neoplastic transformation (Mantovani, Allavena, Sica, & Balkwill, 2008). It has reported that a proinflammatory milieu can also directly increase estrogen production, and then promotes the development of endometrial cancer (Modugno et al., 2005).

In this study, our results observed that rs4851527 and rs2072472 were associated with decreased risk of endometrial cancer, and rs3218896 was significantly associated with an increased risk of endometrial cancer. Previous study reported that the SNP rs4851527 was associated with an increased risk of IgA nephropathy in the over‐dominant model (GA vs. GG‐AA) (Xie et al., 2015). However, association studies indicated that rs4851527 was associated with decreased risk of tuberculosis (Ren et al., 2018) and ankylosing spondylitis (Xia et al., 2015). No significant association was found between the two SNPs (rs3218896 and rs2072472) and risk of IgA nephropathy (Xie et al., 2017), tuberculosis (Ren et al., 2018), and breast cancer (Zuo et al., 2018). Moreover, no significant association was also found between rs2072472 and the risk of ankylosing spondylitis (Xia et al., 2015) and endometriosis (Chun et al., 2012). Previous study reported that rs3218977 was found to be associated with a 0.71‐fold decrease risk of IgA nephropathy in the dominant model (GA‐GG vs. AA) (Xie et al., 2017). A case‐control association study found that the SNP rs3218977 was associated with an increased risk of aggressive periodontitis in the additive model in the Japanese (Kamei et al., 2014). However, rs3218977 was associated with an increased risk of tuberculosis in the dominant model (Ren et al., 2018). However, no significant association was also found between rs3218977 and risk of endometrial cancer in this study. These inconsistencies may be explained by the genetic polymorphism which may have different effects on diseases.

Some potential limitations of the present study should be considered when interpreting the results. First, our result is the first to find that the *IL‐1R2* polymorphisms were associated with the risk of endometrial cancer in the Chinese Han population. Therefore, additional studies are needed to confirm the association between *IL‐1R2 *polymorphisms and endometrial cancer risk with large samples. Second, the potential risk factors such as early menarche, unopposed estrogen, late menopause, and obesity were not analyzed in this study due to the lack of relevant clinical data. More clinical and experimental data were needed to be collected to help us understand better the role of *IL‐1R2 *polymorphisms in the development of endometrial cancer. Third, the biological functions of these SNPs were not analyzed. The expression level of the *IL‐1R2* gene should also be measured in future studies to assess the effects of polymorphisms on *IL‐1R2* gene.

## 5 CONCLUSIONS

In conclusion, the present study results indicated that rs4851527, rs3218896, and rs2072472 in the *IL‐1R2 *gene were associated with endometrial cancer susceptibility in the Chinese Han population. These findings suggested that *IL‐1R2 *polymorphisms may contribute to the development of endometrial cancer. However, further studies are required to confirm the results of the study with a large sample and investigate the detailed mechanisms of the variants that affect *IL‐1R2* gene function.

## CONFLICT OF INTEREST

The authors declare that they have no conflicts of interest.
